# Mechanisms for Vascular Cell Adhesion Molecule-1 Activation of ERK1/2 during Leukocyte Transendothelial Migration

**DOI:** 10.1371/journal.pone.0026706

**Published:** 2011-10-21

**Authors:** Hiam Abdala-Valencia, Sergejs Berdnikovs, Joan M. Cook-Mills

**Affiliations:** Allergy-Immunology Division, Northwestern University Feinberg School of Medicine, Chicago, Illinois, United States of America; Institute of Hepatology London, United Kingdom

## Abstract

**Background:**

During inflammation, adhesion molecules regulate recruitment of leukocytes to inflamed tissues. It is reported that vascular cell adhesion molecule-1 (VCAM-1) activates extracellular regulated kinases 1 and 2 (ERK1/2), but the mechanism for this activation is not known. Pharmacological inhibitors of ERK1/2 partially inhibit leukocyte transendothelial migration in a multi-receptor system but it is not known whether VCAM-1 activation of ERK1/2 is required for leukocyte transendothelial migration (TEM) on VCAM-1.

**Methodology/Principal Findings:**

In this study, we identified a mechanism for VCAM-1 activation of ERK1/2 in human and mouse endothelial cells. VCAM-1 signaling, which occurs through endothelial cell NADPH oxidase, protein kinase Cα (PKCα), and protein tyrosine phosphatase 1B (PTP1B), activates endothelial cell ERK1/2. Inhibition of these signals blocked VCAM-1 activation of ERK1/2, indicating that ERK1/2 is activated downstream of PTP1B during VCAM-1 signaling. Furthermore, VCAM-1-specific leukocyte migration under physiological laminar flow of 2 dynes/cm^2^ was blocked by pretreatment of endothelial cells with dominant-negative ERK2 K52R or the MEK/ERK inhibitors, PD98059 and U0126, indicating for the first time that ERK regulates VCAM-1-dependent leukocyte transendothelial migration.

**Conclusions/Significance:**

VCAM-1 activation of endothelial cell NADPH oxidase/PKCα/PTP1B induces transient ERK1/2 activation that is necessary for VCAM-1-dependent leukocyte TEM.

## Introduction

The transendothelial migration (TEM) of leukocytes is critical for inflammatory responses, immune surveillance, leukocyte homing and mobilization of hematopoietic progenitor cells [1]. The process of TEM involves the sequential rolling and firm adhesion of leukocytes on vascular adhesion molecules followed by the diapedesis of the bound leukocytes [1]. The vascular adhesion molecule VCAM-1 mediates leukocyte rolling and adhesion to endothelium during VCAM-1-dependent eosinophil infiltration into the lung in experimental ovalbumin-induced asthma [2], as well as T-cell infiltration across the blood-brain barrier in experimental allergic encephalomyelitis [3]. VCAM-1-dependent migration is important in vivo because, in several diseases, leukocytes migrate on VCAM-1[4]. Because of this critical role for VCAM-1 in these diseases, targeting of VCAM-1 or its ligand VLA-4 has been used to treat clinical disease [4].

Leukocyte binding to vascular cell adhesion molecule-1 (VCAM-1) triggers signaling events in endothelial cells that are critical during VCAM-1-dependent TEM. We have previously reported that VCAM-1 activates the endothelial cell NADPH oxidase NOX2, which catalyzes the release of low levels of reactive oxygen species (ROS) (1 µM H_2_O_2_) [5], [6]. H_2_O_2_ diffuses through membranes to oxidize and transiently activate endothelial cell-associated protein kinase Cα (PKCα) [7], [8]. PKCα then phosphorylates and activates endothelial cell protein tyrosine phosphatase 1B (PTP1B) [7], [8]. VCAM-1 signals through ROS, PKCα, and PTP1B are required for VCAM-1-dependent leukocyte TEM in vitro [4], [5], [6], [7], [8]. It has been reported that NOX2 and ROS are required for VCAM-1-dependent leukocyte recruitment in vivo [4], [9], [10], [11].

It has also been reported that VCAM-1 ligation activates the serine/threonine kinases extracellular regulated kinases 1 and 2 (ERK1/2) [12] but the mechanism for this activation is not known. It is reported that in cytokine-stimulated primary cultures of endothelial cells, inhibition of ERK1/2 with pharmacological inhibitors, which have additional off-target effects, partially inhibits leukocyte transendothelial migration across the endothelial cells in vitro [12], [13]. Moreover, because the cytokine-stimulated primary endothelial cells express several adhesion molecules that support leukocyte transendothelial migration, it is not known in these studies whether ERK1/2 is involved in VCAM-1-mediated leukocyte transendothelial migration.

In this report, we demonstrate, in primary cultures of human endothelial cells and mouse endothelial cell lines, that VCAM-1 activation of endothelial cell ERK1/2 is mediated by endothelial NADPH oxidase, PKCα and PTP1B. Moreover, inhibition of endothelial ERK2 blocks VCAM-1-dependent leukocyte transendothelial migration.

## Results

### Endothelial cell ERK1/2 is required for VCAM-1-dependent leukocyte migration across endothelial cells

It is reported that pharmacological inhibition of ERK1/2 with PD98059 blocks leukocyte transendothelial migration across endothelial cells that express multiple adhesion molecules [12]. However, it is not known whether VCAM-1-mediated leukocyte transendothelial migration requires ERK1/2 or ERK's classical upstream activator MEK1/2. Therefore, we determined whether endothelial MEK1/2 and ERK2 are required for VCAM-1-dependent leukocyte migration. We used pharmacological inhibitors and dominant negative ERK1/2 approaches to block MEK1/2 or ERK1/2. To examine the function of VCAM-1 signals during VCAM-1-dependent leukocyte transendothelial migration, the endothelial cell line mHEVa was used because it expresses VCAM-1 but not other known adhesion molecules for leukocyte transendothelial migration [4]. It is previously reported that this migration assay is dependent on the constitutively expressed VCAM-1. Blocking with anti-VCAM-1 or anti-VLA4 antibodies immediately before the addition of leukocytes to the endothelial cells inhibits leukocyte adhesion and migration [4], [6], [14], [15]. The VCAM-1-mediated transendothelial migration is also dependent on VCAM-1 signal transduction through NOX2, PKCα, PTP1B, and matrix metalloproteinases and on endothelial cell production of the chemokine MCP-1 [4], [5], [6], [7], [8], [14], [16]. Moreover, antibody crosslinking of VCAM-1 activates these VCAM-1 signals with the same magnitude and time course in the mHEVa cell lines as in primary cultures of endothelial cells [4], [5], [7], [8], [16].

**Figure 1:**
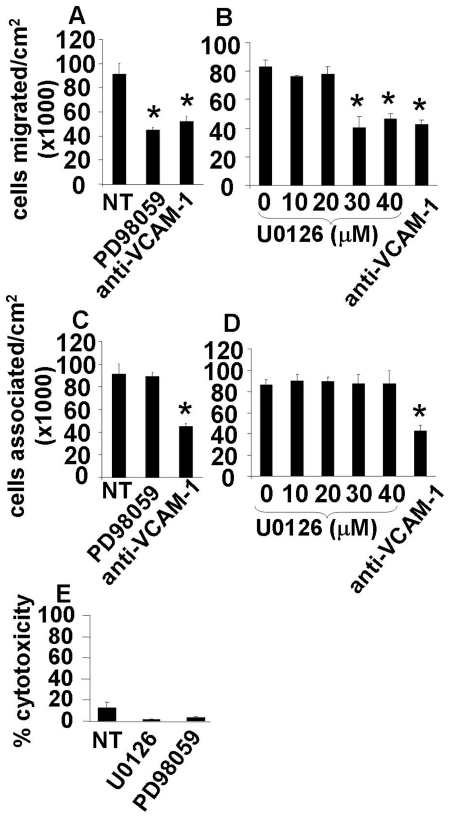
Inhibition of MEK, which is known to activate ERK1/2, blocks VCAM-1-dependent spleen leukocyte TEM. Confluent monolayers of mHEVa cells were nontreated (NT) or treated for 30 minutes with the MEK inhibitors PD98059 (20 µM) or U0126 (20–40 µM). To block leukocyte binding to VCAM-1, the endothelial cells were treated with a blocking anti-VCAM-1 antibody without a secondary crosslinking antibody. **A–D**) Splenic leukocytes were added to the endothelial monolayer, allowed to briefly settle to mediate cell contact and then exposed to 2 dynes/cm^2^ laminar flow for 15 minutes to examine migration (**A and B**) or 2 minutes to examine leukocyte-endothelial cell association (**C and D**). Then, cells were washed and fixed in 3% paraformaldehyde for 1 hour and examined by phase contrast microscopy [7], [8], [65]. Non-migrated leukocytes are phase-light and migrated leukocytes appear as phase-dark [7], [8], [65]. We previously reported that the leukocytes that migrated are >88% lymphocytes as determined by flow cytometry [6]. **E**) Relative cytotoxicity was determined by the G6PDH assay; PD98059 (20 µM) and U0126 (40 µM) were not cytotoxic as compared to the nontreated control cells. Data in each panel are from 3 experiments. *, p<0.05 compared to **A,C**) NT groups, **B,D**) 0 minutes groups, or compared to DMSO-treated or last washes (data not shown). Inhibitors had no effect on cell viability, as determined by Trypan blue exclusion (data not shown).

To examine endothelial MEK1/2 function in VCAM-1-dependent TEM, confluent monolayers of mHEVa cells were nontreated or pretreated for 30 minutes with two inhibitors of MEK1/2, PD98059 or U0126. PD98059 was chosen since reports show that it inhibits activation of inactive MEK1 and MEK 2 with IC50 values of 4 µM and 50 µM, respectively [17], [18], [19]. U0126 irreversibly inhibits both active and inactive MEK1/2 [19]. U0126 has been found to be a selective and highly potent selective inhibitor of MAPK cascade with IC_50_ values of 70 and 60 nM for purified MEK1 and MEK2, respectively [19], [20]. The endothelial cells were pretreated with MEK inhibitors PD98059 (20 µM) or U0126 (10–40 µM) at doses typically used (50 µM) for inhibition of this enzyme in endothelial cells [21], [22]. After treatment with these MEK1/2 inhibitors, the endothelial cells were washed five times. The last wash was added to a set of untreated endothelial cells to demonstrate that effective concentrations of free inhibitor were removed since there was no effect of the last wash on TEM (data not shown). Spleen leukocytes which were >90% lymphocytes were added. After 15 minutes at 2 dynes/cm^2^ laminar flow, the monolayers were washed and fixed; leukocyte transendothelial migration was quantified by phase contrast microscopy [23]. Anti-VCAM-1 blocking antibodies inhibited leukocyte transendothelial migration and leukocyte adhesion (**Figure 1A–D**) but migration was not blocked by isotype control antibodies as we previously reported [5]. The MEK inhibitors did not affect cell viability as determined by trypan blue exclusion (data not shown), did not increase relative cytotoxicity as compared to the nontreated control as determined by the G6PDH release assay (**Figure 1E**) and did not affect the expression of VCAM-1 as determined by flow cytometry (data not shown). PD98059 (20 µM) and U0126 (30–40 µM) significantly inhibited leukocyte transmigration (**Figure 1A,B**). Furthermore, neither PD98059 nor U0126 inhibited leukocyte adhesion (**Figure 1C,D**), indicating that endothelial cell MEK1/2 is not required for leukocyte adhesion but is required for the leukocyte TEM.

**Figure 2:**
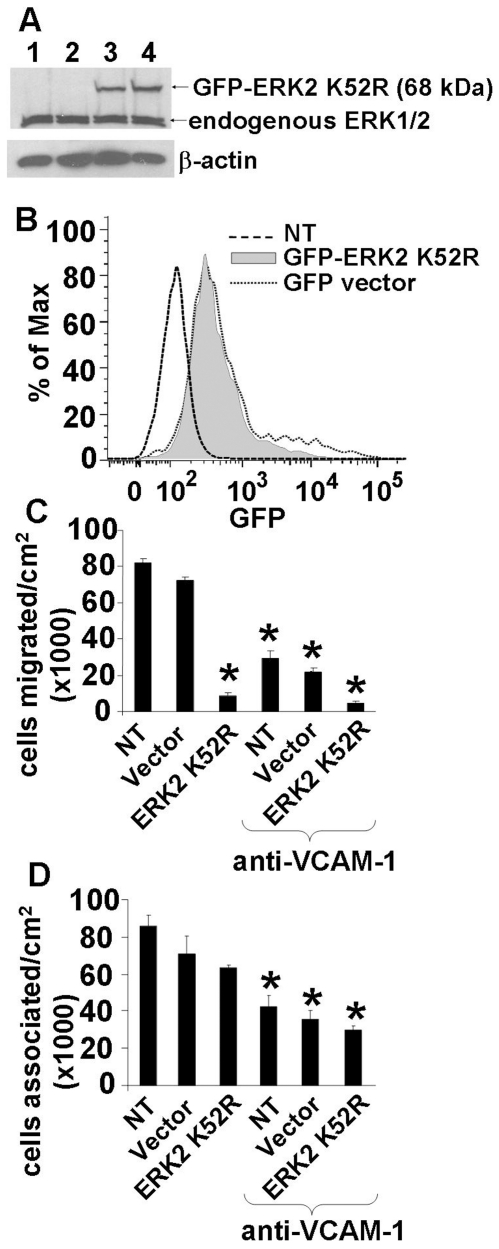
Inhibition of endothelial cell ERK1/2 with an ERK2 dominant negative plasmid (K52R) blocks VCAM-1-dependent leukocyte migration. Two million mHEVa endothelial cells, that were grown to 70∼80% confluence, were suspended with trypsin and transfected with GFP-ERK2 K52R or vector control using the Amaxa nucleofector method. Transfected cells were seeded onto 9 cm^2^ culture slides. Four hours after nucleofection, cells were examined for ERK1/2 expression or used in TEM assays. Greater than 60% of the endothelial cells were transfected as determined by flow cytometry with detection of GFP-ERK2 K52R (data not shown). The transfected endothelial cells formed confluent monolayers in 4 hours and were greater than 85% viable (data not shown). In addition, to block leukocyte binding to VCAM-1, the endothelial cells were treated with a blocking anti-VCAM-1 antibody without a secondary crosslinking antibody. **A**) The endothelial cells were washed with ice-cold phosphate-buffered saline and examined by western blot for ERK1/2 and β-actin expression. Lanes 1 and 2 are two samples of vector-treated cells. Lanes 3 and 4 are two samples of cells treated with GFP-ERK2 K52R. **B**) Leukocyte TEM under laminar flow at 2 dynes/cm^2^, **C**) Leukocyte association assay under laminar flow at 2 dynes/cm^2^. Data for each panel are from 3 experiments. *, p<0.05 compared to non-treated (NT) or vector transfected groups.

It was also determined whether transient transfection of mHEVa cells with the ERK2 dominant negative GFP-ERK2 K52R [24] blocks VCAM-1-dependent leukocyte TEM. The cells expressed the dominant negative ERK2 K52R as examined by western blot (**Figure 2A**). Under our optimized conditions for transfection as described in the methods, GFP-ERK2 K52R-transfected and vector-GFP-transfected mHEVa cells that were cultured in chamber slides for 4 hours had >70% transfection efficiency as analyzed by flow cytometry for GFP (**Figure 2B**). The transfection did not affect mHEVa cell expression of VCAM-1 as determined by immunolabeling and flow cytometry (data not shown). The transfected endothelial cells were plated at a density to form confluent monolayers in 4 hours. The endothelial cells were greater than 85% viable at 4 hours of culture (data not shown). In contrast, since ERK1/2 is a survival signal for endothelial cells [25], [26], [27], [28], [29], [30], at 24 hours of culture the dominant negative ERK1/2 transfected cells began to undergo cell death whereas vector transfected cells survive (data not shown). Therefore, the transient transfection studies were performed at 4 hours when confluent monolayers of viable endothelial cells are formed. At 4 hours post transfection, TEM studies were performed in the parallel plate flow chamber assay at 2 dynes/cm^2^ laminar flow or static conditions for 15 minutes [8]. The transfection with the dominant negative ERK2 K52R inhibited VCAM-1-dependent leukocyte TEM as compared to the vector control under laminar flow (**Figure 2C**) or static conditions (data not shown). Anti-VCAM-1 blocking antibodies inhibited leukocyte transendothelial migration for all the treatments compared to the vector-treated group without anti-VCAM-1 (**Figure 2C**). The dominant negative ERK2 K52R did not alter the number of leukocytes associated with the endothelial cells as compared to the vector-transfected cells under laminar flow conditions (**Figure 2D**) or static conditions (data not shown). Anti-VCAM-1 blocking antibodies inhibited leukocyte association with the endothelial cells for all the treatments compared to the corresponding groups without anti-VCAM-1 (**Figure 2D**). In summary, it is demonstrated for the first time that ERK2 activity is necessary for VCAM-1-dependent leukocyte TEM.

### VCAM-1 activates ERK1/2 downstream of NADPH oxidase, PKCα, and PTP1B in mouse endothelial cell lines that mediate VCAM-1-dependent leukocyte transendothelial migration

Endothelial cell signals are required for VCAM-1-dependent leukocyte transendothelial migration [4]. We have reported that, during transendothelial migration, VCAM-1 activates NADPH oxidase for the generation of superoxide and its metabolite H_2_O_2_; the ROS oxidize and activate PKCα that then activates PTP1B [4]. Therefore, we examined anti-VCAM-1 activation of ERK1/2 under static and laminar flow conditions and then determined whether the ERK1/2 activation is blocked by inhibitors of NADPH oxidase, PKCα, or PTP1B.

**Figure 3:**
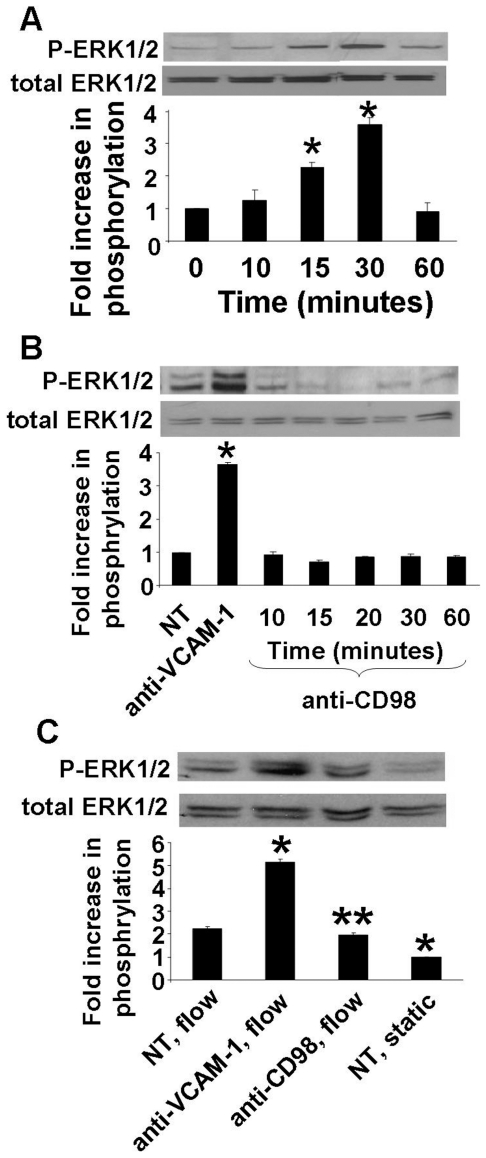
VCAM-1 induces an increase in phosphorylation of ERK1/2 in endothelial cell lines under static and laminar flow conditions. Confluent monolayers of mHEVa cells were stimulated with 27 µg/ml anti-VCAM-1 (or the binding control anti-CD98) plus 15 µg/ml of a secondary antibody. ERK1/2 Thr202/Tyr204 phosphorylation (P-ERK1/2) and total ERK1/2 was determined by western blot. **A**) Time course for anti-VCAM-1 activation of ERK1/2 Thr202/Tyr204 phosphorylation under static conditions. **B**) Stimulation of VCAM-1 for 30 minutes and time course for antibody crosslinking of the control CD98 under static conditions. **C**) Confluent monolayers of HEV were non-treated (NT) or stimulated with anti-VCAM-1 or the control anti-CD98 under 2 dynes/cm^2^ laminar flow or nontreated under static conditions for 15 minutes. *, p<0.05 compared to **A**) 0 minutes, **B**) NT and **C**) the NT,flow group. In panel **C**) **, p<0.5 compared to the anti-VCAM-1, flow group.

To examine the time course for anti-VCAM-1 activation of ERK1/2 under static and laminar flow conditions, confluent monolayers of mHEVa cells were stimulated with a confluent monolayer of anti-VCAM-1 antibody-coated beads for 10 to 60 min. Antibody crosslinking of VCAM-1 mimics physical leukocyte interaction with endothelial cells for the activation of VCAM-1-dependent signals [5], [6], [7], [8], [16]. The negative control included treatment with a confluent monolayer of anti-CD98 antibody-coated beads for 10 to 60 min, since CD98 is expressed by mHEVa cells, but does not signal through ERK1/2 [31]. After stimulation with antibody-coated beads, the cells were examined by Western blot for phosphorylation of ERK1/2 Thr202/Tyr204, the active form of ERK1/2. Antibody crosslinking of VCAM-1 under static conditions induced an increase in ERK1/2 phosphorylation at 15–30 minutes in mHEVa cells (**Figure 3A**). VCAM-1 stimulation did not increase total ERK1/2 expression (**Figure 3A–B**). There was no increase in ERK1/2 phosphorylation with the negative control anti-CD98 coated beads (**Figure 3B**). To study the effect of laminar flow on ERK1/2 activation, confluent monolayers of endothelial cells were assembled in parallel plate flow chambers, anti-VCAM-1-coated beads or anti-CD98-coated beads were added to the flow chamber and then laminar flow at 2 dynes/cm^2^ was applied for 15 minutes. Equivalent numbers of the anti-VCAM-1-coated beads or anti-CD98-coated beads were loaded onto the endothelial cells and were bound to the endothelial cells (data not shown). To avoid stimulation of the endothelial cells by serum growth factors in fresh culture medium, we perfused the laminar flow chambers with media taken from cultured mHEVa cells. ERK1/2 Thr202/Tyr204 phosphorylation was significantly increased when mHEVa cells were stimulated with anti-VCAM-1 beads, compared to nontreated or the anti-CD98 binding controls under flow conditions (**Figure 3C**). When we compared nontreated endothelial cells under static conditions with nontreated endothelial cells under flow conditions, there was a significant but modest increase in ERK1/2 Thr202/Tyr204 phosphorylation in nontreated cells under laminar flow (**Figure 3C**), consistent with previous reports that laminar flow induces some ERK1/2 activation [32], [33]. Anti-VCAM-1 under flow induced a greater increase in ERK1/2 phosphorylation compared to stimulation under static conditions (5 fold increase under flow versus 3 ½ fold increase under static conditions) (**Figure 3**). Together, these data suggest that VCAM-1 stimulates an increase in endothelial ERK1/2 activity under both static and laminar flow at 2 dynes/cm^2^.

**Figure 4:**
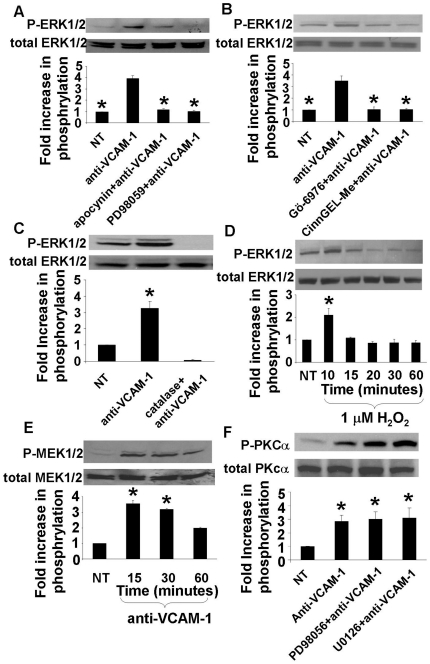
Mechanisms for VCAM-1 activation of ERK1/2 in endothelial cell lines. Monolayers of mHEVa cells were nontreated (NT) or incubated for 30 minutes with apocynin (4 mM), PD98059 (30uM), U0126 (40 µM), Gö-6976 (2.3 nM), CinnGEL 2-methylester (10 µM), or catalase (5000 U/ml) where indicated. These are the optimal doses for these inhibitors as we have previously described [5], [7], [8]. At these concentrations, none of the inhibitors had any significant effects on the basal level of ERK1/2 in the absence of anti-VCAM-1 stimulation (data not shown). After treatement with the inhibitor, the endothelial cells were stimulated with anti-VCAM-1 antibody plus a secondary antibody to crosslink VCAM-1 for 30 min under static conditions. We examined phosphorylation of **A,B,F**) ERK1/2 Thr202/Tyr204 (P-ERK1/2), **C**) PKCα Thr638 (P-PKCα), or **D**) MEK1/2 Ser217/221 (P-MEK1/2) by western blot. **E**) mHEVa cells were treated with exogenous 1 µM H_2_O_2_ for 10–60 minutes and ERK1/2 Ser217/221 phosphorylation was determined by western blot. The phosphorylation status of ERK1/2 Thr202/Tyr204, PKCα Thr638, or MEK1/2 Ser217/221 is presented as the fold increase in the ratio of the relative intensity of the phosphorylated enzyme to total ERK1/2, total PKCα or total MEK1/2 expression. Representative western blots are shown and data are presented as the mean ± standard deviation from 3 experiments. (**A**, **B**) *, p<0.05 less than anti-VCAM-1-treated group. (**C–F**) *, p<0.05 greater than NT.

Then, we determined whether ERK1/2 is activated by VCAM-1′s signaling cascade (NADPH oxidase, 1 µM H_2_O_2_, PKCα and PTP1B). Anti-VCAM-1 activation of ERK1/2 Thr202/Tyr204 phosphorylation in mHEVa cells under static conditions was blocked by the NADPH oxidase inhibitor apocynin or scavenging extracellular ROS with catalase (**Figure 4A, C**), suggesting that ERK1/2 functions downstream of NADPH oxidase. In the absence of VCAM-1 stimulation, apocynin and catalase did not alter background ERK1/2 phosphorylation (data not shown). Since we previously reported that binding to VCAM-1 stimulates endothelial cell NADPH oxidase, resulting in the generation of 1 µM H_2_O_2_ [5] and that exogenous addition of 1 µM H_2_O_2_ is sufficient for the activation of PKCα and PTP1B in endothelial cells at 10 minutes [7], [8], it was determined whether exogenous 1 µM H_2_O_2_ activated endothelial cell ERK1/2. At 10 minutes, 1 µM H_2_O_2_ significantly increased ERK1/2 Thr202/Tyr204 phosphorylation in mHEVa cells (**Figure 4D**). Therefore, exogenous ROS, at concentrations that are generated by VCAM-1-outside-in signals [6], stimulate a significant increase in ERK1/2 phosphorylation. Anti-VCAM-1 activation of ERK1/2 Thr202/Tyr204 phosphorylation in mHEVa cells was also blocked by the PKCα inhibitor Gö-6976, and the PTP1B inhibitor CinnGEL 2-methyl ester (**Figure 4B**). At these concentrations, none of the inhibitors had any significant effects on the basal level of ERK1/2 in the absence of anti-VCAM-1 stimulation (data not shown). The inhibitors used at these optimal doses did not affect cell viability as we previously reported [**7]**, [8****].

Since ERK1 and ERK2 are classically activated through MEK1 and MEK2-mediated phosphorylation of ERK's activation loop residues Thr202/Tyr204 on ERK1 and Thr185/Tyr187 on ERK2 [34], we determined whether MEK1/2 mediated VCAM-1 activation of ERK1/2. In confluent monolayers of mHEVa cells, antibody crosslinking of VCAM-1 significantly increased serine 217/221 phosphorylation of MEK1/2 at 15 to 30 minutes as compared to nontreated cells (**Figure 4E**); this time course is similar to the 15–30 minutes for activation of ERK1/2 in **Figure 3A**. The MEK1/2 inhibitor PD98059 blocked VCAM-1-activated phosphorylation of ERK1/2 (**Figure 4A**) and leukocyte transendothelial migration (**Figure 1A,C**). In contrast, inhibition of MEK1/2 with PD98059 or U0126 did not block activation of PKCα (**Figure 4F**), indicating that MEK1/2 and ERK1/2 function downstream of PKCα during VCAM-1 signaling. Together, the data indicate that, during VCAM-1 signaling, ERK1/2 functions downsteam of NADPH oxidase, PKCα, and PTP1B in mouse endothelial cell lines that mediate VCAM-1-dependent leukocyte transendothelial migration.

### VCAM-1 activates ERK1/2 downstream of NADPH oxidase, PKCα, and PTP1B in primary cultures of human endothelial cells

**Figure 5:**
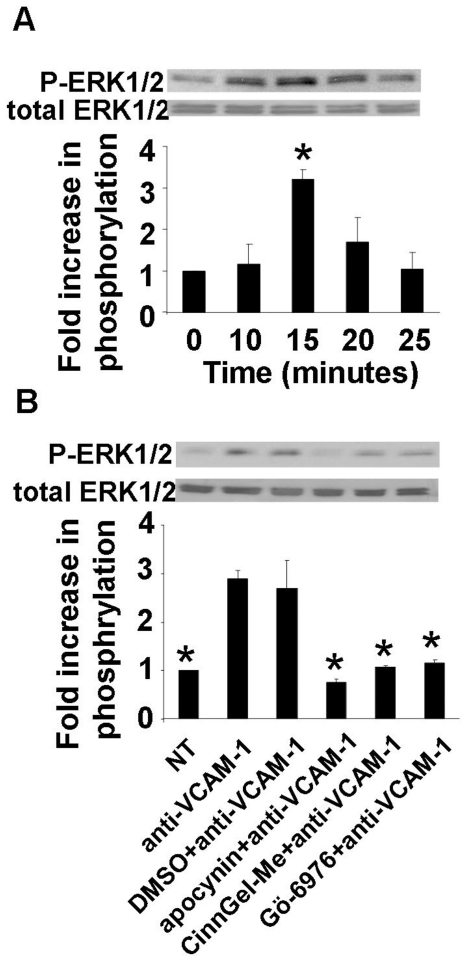
Mechanisms for VCAM-1 activation of ERK1/2 in HMVEC-L. Treatment of HMVEC-L cells overnight with 10ng/ml TNF-α induced VCAM-1 expression (data not shown). **A**) Confluent monolayers of TNF-α-treated HMVEC-Ls were nontreated (NT) or treated with 27 µg/ml anti-VCAM-1 plus 15 µg/ml of a secondary antibody to crosslink and stimulate VCAM-1. Phosphorylation of ERK1/2 Thr202/Tyr204 (P-ERK1/2) and total expression of ERK1/2 was examined by western blot using rabbit anti-phospho ERK1/2 Thr202/Tyr204 (1/1000) followed by HRP-conjugated anti-rabbit (1/2000) and ECL detection. **B**) Confluent monolayers of TNF-α stimulated HMVEC-L cells in 12 well plates were nontreated or incubated for 30 minutes with the solvent control DMSO, apocynin (4 mM), Gö-6976 (2.3 nM) or CinnGEL 2-methylester (10 µM). These endothelial cells were then stimulated with anti-VCAM-1 antibody plus a secondary antibody for 15 minutes. The apocynin, DMSO, Gö-6976 or CinnGEL 2-methylester had no effect on endothelial cell viability as determined by trypan blue exclusion and had no effect on VCAM-1 expression as determined by flow cytometry (data not shown). Representative western blots are shown. Data presented are the mean ± standard deviation from 3 experiments. The phosphorylation status of ERK1/2 is presented as the fold increase in the ratio of the relative intensity of P-ERK1/2 divided by the relative intensity of the loading control (total ERK1/2). *, p<0.05 compared to **A**) NT cells or **B**) anti-VCAM-1 stimulated cells.

We have reported that, in primary cultures of human endothelial cells, VCAM-1 signals through NADPH oxidase, PKCα and PTP1B [7], [8]. Therefore, we determined whether VCAM-1 activates ERK1/2 downstream of NADPH oxidase, PKCα, and PTP1B in primary cultures of human microvascular endothelial cells from the lung (HMVEC-L). For these studies, HMVEC-Ls were treated overnight with 10 ng/ml TNFα to induce expression of several adhesion molecules, including VCAM-1 and ICAM-1 [7], [8], [35], [36]. TNFα induced expression of VCAM-1 by HMVEC-L cells as determined by immunolabeling and fluorescence microscopy (data not shown). Therefore, since TNFα induces expression of several adhesion molecules, VCAM-1 on TNFα-treated HMVEC-Ls was specifically activated by crosslinking with anti-VCAM-1 plus a secondary antibody for 10 to 30 min and then analyzed by Western Blot for Thr202/Tyr204 phosphorylation of ERK1/2. Stimulation of VCAM-1 at 15 minutes induced Thr202/Tyr204 phosphorylation of ERK1/2 in TNFα -pretreated cultures of HMVEC-L cells without altering total ERK1/2 (**Figure 5A, B**). This time point for human VCAM-1 activation of ERK1/2 in human endothelial cells is generally consistent with the time course for mouse VCAM-1 activation of ERK1/2 in the murine endothelial cell lines, even though the anti-human VCAM-1 and anti-mouse VCAM-1 antibodies differ. In addition, the time course for VCAM-1 activation of ERK1/2 in HMVEC-Ls is consistent with the time course for VCAM-1 signaling in HMVEC-Ls including maximal stimulation of PKCα at 10 minutes and PTP1B at 15 minutes [7], [8]. To determine whether ERK1/2 functions downstream of NADPH oxidase, PKCα and PTP1B during VCAM-1 signaling, TNF-α stimulated HMEVC-L cells were treated with the vehicle control 0.1% DMSO, the NADPH oxidase inhibitor apocynin (4 mM), the PKCα inhibitor Gö-6976 (2.3 nM) or the PTP1B inhibitor CinnGEL 2-methylester (10 µM) for 30 min and then stimulated with anti-VCAM-1 plus secondary antibody for 15 min. The inhibitors were used at the optimal doses and they did not affect endothelial cell viability as we previously reported [5], [7], [8]. None of the inhibitors had any significant effects on the basal level of ERK1/2 protein expression in the absence of anti-VCAM-1 as determined by western blot (data not shown). Anti-VCAM-1-activated Thr202/Tyr204 phosphorylation of ERK1/2 was blocked by inhibitors of NADPH oxidase, PTP1B and PKCα (**Figure 5B**). Together, these data indicate that VCAM-1 activates ERK1/2 downstream of NADPH oxidase, PKCα and PTP1B in mouse endothelial cell lines (mHEVa cells) and primary cultures of endothelial cells (HMVEC-L cells). This is the first report for a mechanism for VCAM-1 activation of ERK1/2.

## Discussion

In this manuscript, we report that VCAM-1 activation of ERK1/2 participates in VCAM-1-dependent leukocyte transendothelial migration and identify a mechanism for VCAM-1 activation of ERK1/2 in endothelial cells. VCAM-1-activated ERK2 activity is necessary for VCAM-1-dependent leukocyte migration across mHEVa cells, because migration is inhibited by anti-VCAM-1 blocking antibodies [5], [6], by the ERK1/2 inhibitors PD98059 or U0126, and by transient transfection of endothelial cells with the dominant negative ERK2 K52R. Furthermore, endothelial ERK1/2 functions downstream of VCAM-1-activated NADPH oxidase, because the inhibitor apocynin blocked anti-VCAM-1-dependent ERK1/2 activation and we have previously reported that VCAM-1 does not signal through other ROS-generating enzymes [4]. ROS were sufficient for activation of ERK1/2 since exogenous addition of 1 µM H_2_O_2_, which corresponds to the level of H_2_O_2_ produced during VCAM-1 signaling [6], [15], increased ERK1/2 activation with about the same magnitude and time course as anti-VCAM-1. Moreover, we report that ERK1/2 activation was dependent on VCAM-1-activated PKCα [8] and PTP1B [7] in human primary endothelial cells and mouse endothelial cell lines because inhibition of PKCα and PTP1B blocked VCAM-1 activation of ERK1/2. Thus, VCAM-1 binding increased Thr202/Tyr204 phosphorylation of ERK1/2 in mouse endothelial cell lines and in human primary cultures of endothelial cells and this activation was downstream of VCAM-1 activation of NADPH oxidase, PKCα, or PTP1B. This is the first study that identifies a mechanism for VCAM-1 activation of ERK1/2 during leukocyte TEM.

It has been reported that endothelial cell ERK1/2 functions in leukocyte TEM since pretreatment of endothelial cells with a MEK1/2 inhibitor, PD98059, attenuates eosinophil transmigration across primary cultures of HUVECs that express several adhesion molecules [12], [37]. Since several adhesion molecules were expressed by the endothelial cells, it is not known in their studies whether ERK1/2 was required for VCAM-1-mediated leukocyte TEM. Moreover, this MEK1/2 inhibitor PD98059, has been demonstrated to have off target effects of inhibition of voltage-dependent Ca2+ channels [38] and we have reported that VCAM-1 signals through Ca2+ channels [15]. Besides our pharmacological approach demonstrating that two MEK inhibitors U0126 and PD98059 block VCAM-1-dependent leukocyte TEM, we report, using a molecular approach, that VCAM-1 activation of ERK1/2 is required for VCAM-1-dependent TEM since transient transfection of endothelial cells with dominant negative ERK2 K52R reduced leukocyte TEM on VCAM-1. Thus, VCAM-1-activated ERK1/2 is an obligatory signal in VCAM-1-dependent TEM.

It has been reported that VCAM-1 ligation in large vessel endothelial cells (human umbilical vein endothelial cells, HUVECs) induces an increase in ERK2 activity [12] but they did not report a mechanism for activation. They showed that VCAM-1 activated ERK2 under laminar flow but not static conditions [12]. In contrast, we demonstrate that anti-VCAM-1-coated beads stimulate an increase in ERK1/2 phosphorylation under both laminar flow or static conditions in mHEVa cells or HVMEC-L cells without adding fresh media. In our studies, under the flow conditions of 2 dynes/cm^2^, ERK1/2 phosphorylation was amplified as compared to static conditions. It is possible that in the previous report [12], they did not detect a VCAM-1-induced activation of ERK2 under static conditions as a result of differences in serum conditions, static/flow conditions, or differences in endothelial cell sources (microvascular endothelial cells versus large vessel HUVECs).

It has been reported that there is increased activity of MAPKs with ligation of ICAM-1, VCAM-1 or E-selectin ligation in epithelial [39], [40], [41] and endothelial cells [12], [42], [43], [44], [45]. Adherence of neutrophils or ICAM-1 crosslinking activates ERK in endothelial cells [42], [43], [46], [47], [48], epithelial cells [39], [40], [49], [50], and smooth muscle cells [13]. Moreover, ligation of E-selectin leads to shear-dependent ERK1/2 phosphorylation in HUVECs [12], [44]. Thus, several adhesion molecules activate ERK/12 in different cell types. In this study, we report a mechanism for VCAM-1 activation of ERK1/2 via activation of NADPH oxidase, PKCα and PTP1B in microvascular mouse endothelial cell lines and human microvascular endothelial cells. In contrast, crosslinking of another endothelial cell surface molecule, CD98, does not activate ERK1/2, indicating that VCAM-1 activation of ERK is specific.

Others report that endothelial cell ERK1/2 or p38 MAPK activation can occur downstream of intracellular oxidant generation [45], [51], [52], [53]. For example, oxygen-derived free radicals were previously reported to induce the activation of ERKs and p38 MAPKs in cultured cardiac myocytes [54]. Receptors other than VCAM-1 have been reported to activate ERK1/2 through reactive oxygen species (ROS) in endothelial cells [51], [52], [53] or PKC-dependent signaling pathways in epithelial cells and T cells [41], [50], [55], [56], [57], [58]. Whether there is crosstalk between these adhesion molecules through PKCα and ERK1/2 is not known. Regarding adhesion molecule signaling crosstalk, there may be crosstalk since both VCAM-1 and ICAM-1 signal through PKCα[4]. However, the adhesion molecule signaling pathways differ since VCAM-1 signals through NADPH oxidase whereas ICAM-1 signals through xanthine oxidase [4], [59]. Moreover, the adhesion molecule PECAM-1 does not signal through reactive oxygen species [5]. Thus, there are unique signals for these adhesion molecules as well as potential points for crosstalk between some of the adhesion molecules. In this report, we demonstrate that low levels of VCAM-1-stimulated ROS generation, which activate PKCα and PTP1B, induced signals for phosphorylation of ERK1/2 in mouse endothelial cells (mHEVa) and human endothelial cells (HMVEC-Ls). This is consistent with our previous studies demonstrating that pharmacological or molecular approaches to inhibit VCAM-1-induced signals through ROS, PKCα and PTP1B block leukocyte TEM in vitro and block VCAM-1-dependent leukocyte recruitment in vivo [4], [5], [7], [8], [16].

In summary, the data in this report demonstrate that ERK1/2 is a distal signaling intermediate in the VCAM-1-induced NADPH oxidase/PKCα/PTP1B signal transduction pathway and that the activation of ERK1/2 is required for VCAM-1-dependent leukocyte TEM. Thus, VCAM-1 activates the following signaling cascade to support TEM: VCAM-1 activates endothelial cell NADPH oxidase to generate ROS [4], [5]; these ROS transiently activate PKCα [4], [8]; PKCα activity then transiently activates PTP1B [4], [7]; the PTP1B activates signals that then induce an increase in Thr202/Tyr204 phosphorylation of ERK1/2.

## Materials and Methods

### Cells

Human microvascular endothelial cells from the lung (HMVEC-Ls) (CC-Lonza, Walkersville, MD) were grown in EGM-MV endothelial growth medium plus 5% FCS (catalog #CC-3125, Lonza) and were used at passage 2–6. The high endothelial venule-like cell line mHEVa cells was previously derived from BALB/c mouse axillary lymph nodes and cultured as described [60]. The mHEVa cells have been spontaneously immortalized but are not transformed and are constitutively activated with expression of VCAM-1 and MCP-1 [60]. The mHEV cells are an endothelial cell model for examining VCAM-1 function during transendothelial migration. For leukocytes, single cell suspensions were obtained from spleens of male 6-8 week old BALB/c mice (Jackson Laboratories) as previously described ^4^ and the red blood cells were lysed by hypotonic shock [60]. Spleens are used because they are an abundant source of leukocytes and the spleen is contiguous with the blood. The animal procedures were reviewed and approved by the Animal Care and Use Committee at Northwestern University.

### Reagents

Rat anti-mouse VCAM-1 (clone MVCAM.A), mouse anti-human VCAM-1 (clone 51-10C9), and rat anti-mouse CD98 (clone H202-141) were from BD PharMingen, San Diego, CA. Goat anti-mouse IgG1 (catalog #1070-01) and goat anti-rat IgG (catalog #3050-01) were from Southern Biotech, Birmingham, AL. The phospho-p44/42 MAPK (Erk1/2) (Thr202/Tyr204) (clone D13.14.4E) XP™ rabbit antibodies (catalog #4370), the p44/42 MAPK (Erk1/2) (clone 137F5) rabbit antibodies (catalog #4695), the rabbit anti-phospho-MEK1/2 (Ser217/221) (clone 41G9) antibodies (catalog #9154), the rabbit anti-MEK1/2 antibodies (catalog #9122), the HRP-conjugated goat anti-rabbit IgG antibody (catalog #7074), the HRP-conjugated horse anti-mouse IgG, antibody (catalog #7076) and the MEK1/MEK2 inhibitor U0126 (catalog #9903) were from Cell Signaling Technology, Beverly, MA. Apocynin (catalog #102420250) was from Acros Organics, Pittsburg, PA. Gö-6976 (catalog #EI-269) was obtained from Biomol, Plymouth Meeting, PA. H_2_O_2_ (catalog #H-1009) was from Sigma. PD98059 (catalog #BML-EI360-0005) and CinnGEL 2-methylester (catalog #BML-PR114-0001) were purchased from Enzo Life Sciences International, Inc (Plymouth Meeting, PA).

### Transient transfection

The rat ERK2 wild type (WT) sequence containing plasmid pcDNA3-HA-ERK2 WT was purchased from Addgen (plasmid #8974). ERK2 WT sequence was subcloned to pmaxFP-Green-C vector (catalog #VDF-1011, Lonza) using BamHI and KpnI restriction sites. The plasmid was mutated at K52R using Agilent technologies site directed mutagenesis kit (catalog # 200523-5) using the primer GTTCGAGTTGCTATCAGGAAAATCAGTCCTTTTGAGC.

The Nucleofector™ Kit for Human Microvascular Endothelial Cells - Lung (HMVEC-L) (catalog #VPB-1003, Lonza) was used for nucleofection of mHEVa cells. The conditions for optimal transfection efficiency of >70% were as follows: mHEVa cells were plated and grown to 70–80% confluent in 3 days. Since the nucleofection reagent is positively charged and the endothelial cell surface is highly positively charged, the endothelial cells were briefly trypsin-treated to remove some surface charge and to suspend the cells. One million of these endothelial cells were centrifugation at 700 rpm for 10 minutes and all supernatant was carefully removed. The resulting cell pellets were suspended in 100 µl of the nucleofection reagent plus 4 µg pmaxFP-Green-C vector or 4 µg of pmaxFP-Green-C vector containing the ERK2 K52R plasmid. Cells were subsequently transferred into nucleofection cuvettes (Lonza). The endothelial cells were nucleofected by using the S-005 program in the Amaxa Nucleofector II (Lonza). Pre-warmed 500 µl EGM-MV plus 5% FCS culture media was immediately added to the transfected cells, which were subsequently cultured in the same media at a density to form confluent monolayers. The endothelial cells formed confluent monolayers in four hours and were >90% viable. At four hours of culture, transfection was confirmed by western blot for total ERK1/2 and flow cytometry for GFP. Transfection efficiency was determined as a percentage of GFP-positive cells by flow cytometry.

### In vitro cell association and migration assays under laminar flow or static conditions

A parallel plate flow chamber was used to examine leukocyte migration under conditions of laminar flow at 2 dynes/cm^2^, as previously described [7], [8] or static conditions. Spleen cells were used as a source of leukocytes contiguous with the blood stream that could then migrate across endothelial cells. Spleen cell migration across the mHEV cell lines is stimulated by mHEV cell constitutive production of the chemokine MCP-1 [14] and is dependent on adhesion to VCAM-1 and not other known adhesion molecules [5], [60], [61]. We have previously reported that, consistent with the populations isolated from the spleen, after migration across the mHEV cells, the spleen cells are 65–70% B cells, 12–15% CD4+ cells, and 5–8% CD8+ cells [6]. For this migration assay, endothelial cells were grown to confluence on slides and then the slide was placed in a parallel plate flow chamber [23]. In vivo, in the absence of inflammation, the rapid fluid dynamics of the blood result in blood cells located midstream of the vascular flow [62]. However, during inflammation, there is a change of fluid dynamics [62], [63], [64]. With inflammation, vascular permeability increases yielding fluid flow from the blood into the tissues which contributes to bringing blood cells into contact with the endothelium (“margination”) [62], [64]. There is also cell contact as the blood cells leave the capillaries and enter the small diameter postcapillary venules [63]. Therefore, spleen cells (3×10^6^) were added to the flow chamber (3.5 cm^2^) at 2 dynes/cm^2^. Next, to briefly initiate spleen cell contact with the endothelial cells in vitro, the spleen cells were allowed to settle in the chamber as monitored by microscopy and then 2 dynes/cm^2^ was applied for the 15 min laminar flow assay. We have observed by microscopy that during the assay under laminar flow, the spleen cells in contact with the endothelial cells either roll, roll and detach, or roll, firmly attach, and migrate. After cell contact, the focus of the studies is on mechanisms of transendothelial migration under conditions of laminar flow. For this assay, the coculture was exposed to laminar flow at 2 dynes/ cm^2^ at 37°C for 2 min to examine cell association or for 15 min to examine cell migration. After the 2 or 15 min at 2 dynes/ cm^2^, the cells are washed with PBS supplemented with 0.2 mM CaCl_2_ and 0.1 mM MgCl2 because cations are required for cell adhesion. These cells were fixed with 3% paraformaldehyde for 1 h. To quantify migrated spleen cells at 15 min, phase contrast microscopy was used to count migrated cells that are phase dark [65]. It has been reported that the transendothelial migration of an individual leukocyte, after it has rolled to a site of migration, occurs in 2 min [60]. However, transendothelial migration of leukocytes is asynchronous. In the laminar flow assay, spleen cell migration is detected by 15 min. The number of spleen cells that were associated but not migrated (phase light cells) at 15 min is low because in 15 min, the majority of nonmigrating cells roll off the monolayer of endothelial cells as determined by microscopy (data not shown). Therefore, the numbers of spleen cells associated with the endothelial cells at 2 min of laminar flow are those cells that mediated cell-cell contact.

### Antibody coated-beads

For anti-mouse VCAM-1-coated beads, streptavidin-coated 9.9 µm diameter beads (80 µl) (Bangs Laboratories) were labeled with 24 µg of biotin-conjugated goat anti-rat Ig in 375 µl of PBS with gentle rocking for 1 h at 4°C and then washed three times [5]. These beads were incubated with 16 µg of rat anti-mouse VCAM-1 or a rat isotype control antibody in 375 µl of PBS with gentle rocking for 1 h at 4°C and then washed.

### Western blotting

Cell lysates were analyzed by 10% SDS-PAGE and transferred to nitrocellulose membranes by the semi-dry method according to manufacturers instructions (Bio-Rad). The membranes were blocked in 5% non-fat dried milk in Tris-buffered saline plus 0.1% Tween 20 (TBS-T) for 1 hour at room temperature and washed 3 times for 5 minutes in TBS-T. Membranes were incubated with primary antibodies in TBS-T plus 5% milk overnight, washed 3 times for 5 minutes in TBS-T, incubated with anti-rabbit secondary antibodies in TBS-T plus 5% milk for 1 hour, washed 3 times for 10 minutes in TBS-T, and examined for detection with the enhanced chemiluminescence kit (catalog #RPN2132, Amersham) and autoradiography. Equal protein loading was verified by stripping the membrane with Restore Western Blot Stripping Buffer (Pierce, Rockford, IL) for 15 minutes and then labeling with rabbit anti-total ERK1/2 or MEK1/2 where indicated. Densitometry was performed using Image J software (NIH). The data were presented as the fold increase in the ratio of relative intensity of the band / the relative intensity of band for the loading control (total ERK1/2 or total MEk1/2 where indicated).

### Statistics

Data were analyzed by a one way ANOVA followed by Tukey's multiple comparisons test (SigmaStat, Jandel Scientific, San Ramon, CA).
